# Identification of a New Broadly Cross-reactive Epitope within Domain III of the Duck Tembusu Virus E Protein

**DOI:** 10.1038/srep36288

**Published:** 2016-11-08

**Authors:** Chenxi Li, Xiaofei Bai, Runze Meng, Wulin Shaozhou, Qingshan Zhang, Ronghong Hua, Jyung-Hurng Liu, Ming Liu, Yun Zhang

**Affiliations:** 1 State Key Laboratory of Veterinary Biotechnology, Harbin Veterinary Research Institute of Chinese Academy of Agricultural Sciences, Harbin, 150001, China; 2 Institute of Genomics and Bioinformatics, National Chung Hsing University. Taiwan

## Abstract

In 2010, a pathogenic flavivirus termed duck Tembusu virus (DTMUV) caused widespread outbreak of egg-drop syndrome in domesticated ducks in China. Although the glycoprotein E of DTMUV is an important structural component of the virus, the B-cell epitopes of this protein remains uncharacterized. Using phage display and mutagenesis, we identified a minimal B-cell epitope, ^374^EXE/DPPFG^380^, that mediates binding to a nonneutralizing monoclonal antibody. DTMUV-positive duck serum reacted with the epitope, and amino acid substitutions revealed the specific amino acids that are essential for antibody binding. Dot-blot assays of various flavivirus-positive sera indicated that EXE/DPPFG is a cross-reactive epitope in most flaviviruses, including Zika, West Nile, Yellow fever, dengue, and Japanese encephalitis viruses. These findings indicate that the epitope sequence is conserved among many strains of mosquito-borne flavivirus. Protein structure modeling revealed that the epitope is located in domain III of the DTMUV E protein. Together, these results provide new insights on the broad cross-reactivity of a B-cell binding site of the E protein of flaviviruses, which can be exploited as a diagnostic or therapeutic target for identifying, studying, or treating DTMUV and other flavivirus infections.

Flaviviruses are positive-sense RNA viruses classified in the genus *Flavivirus*, family Flaviviridae^1^, which include several important vector-borne viruses of zoonotic nature. Many flaviviruses play a considerable role in human health and disease, such as dengue virus (DENV), West Nile virus (WNV), Japanese encephalitis virus (JEV), and Zika virus (ZIKV). In April 2010, a severe outbreak of a duck viral infection, which led to a devastating drop in egg production (i.e. egg-drop syndrome), spread throughout the major duck-producing regions in China. Postmortem examination of infected ducks indicated severe ovarian hemorrhage, ovaritis, and regression. Genomic sequencing revealed that the outbreak was caused by the duck Tembusu virus (DTMUV), which is a mosquito-borne Ntaya group flavivirus^2^,^3^,^4^,^5^,^6^,^7^.

The DTMUV genome, like that of other flaviviruses, encodes three structural proteins (C, prM/M, and E) and seven nonstructural proteins (NS1, NS2A, NS2B, NS3, NS4A, NS4B, and NS5)^1^,^3^,^8^. In most flaviviruses, the glycosylated E protein is located on the virion surface and plays an important role in virus receptor binding, host-cell entry, and antigenicity^9^. The flavivirus E protein contains three structurally distinct domains: DI, DII, and DIII. The DI domain is composed predominantly of type-specific nonneutralizing epitopes^9^. The DII domain contributes to virus-mediated membrane fusion and contains many cross-reactive epitopes, eliciting neutralizing and nonneutralizing antibodies^9^,^10^,^11^,^12^,^13^. The DIII domain contains multiple type- and subtype-specific epitopes, which elicit only virus-neutralizing antibodies^10^,^14^,^15^,^16^. Although the biologic characteristics of most flaviviruses are well defined, no specific antiviral drugs are available for clinical use against flavivirus infections.

Studies on the immunopathogenesis of severe dengue fever suggest that induction of cross-reactive nonneutralizing antibodies may increase the likelihood of acute disease during subsequent infections with different serotypes^17^,^18^,^19^. Preexisting cross-reactive antibodies form nonneutralized complexes that allow the virus to enter Fc receptor–expressing cells more efficiently. Therefore, the presence of cross-reactive nonneutralized antibodies most likely plays a role in antibody-dependent enhancement of the infection process. Manipulation of a virus to limit antigen exposure and dominant recognition of cross-reactive nonneutralizing sites, while augmenting the induction of protective neutralizing antibodies directed to epitopes, is a useful strategy for vaccine development.

In this study, we performed phage display and structure modeling to map a new epitope within the DIII domain of the DTMUV E protein that generates broad cross-reactivity to other flavivirus-positive sera. These findings better our understanding of the structure–function relations of E protein epitopes that are important for flavivirus biology, which can lead to improved sero-diagnosis, inform vaccine design, and knowledge of flavivirus pathogenesis.

## Results

### Epitope prediction

To map the location of the epitope within the DTMUV E protein, we screened a phage-displayed 12-mer random peptide library using the monoclonal antibody (mAb) 3B6. After three rounds of biopanning, we selected and evaluated several phage clones for their reactivity with the 3B6 mAb and with the negative control anti-porcine interferon-c (IFN-c) mAb. Of 30 clones, 26 (B1–B26) reacted with the 3B6 mAb (OD_450_ ≥ 1.20), whereas the remaining clones were less reactive (OD_450_ <  0.36). None of the selected clones reacted with the IFN-c mAb (OD450 <  0.27) (Supplementary Figure S1). Sequencing of the phage clones with the highest OD values revealed the consensus sequence EXE/DPPFG (Table 1). This amino acid sequence is identical to amino acids 374 to 380 (EVEPPFG) of the E protein of DTMUV.

### Mapping of the minimal epitope

**Figure 1:**
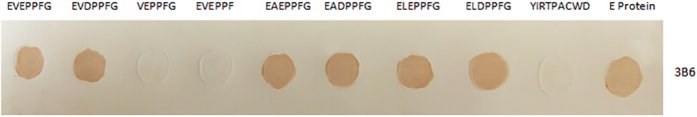
Identification of the E protein epitope. Peptide fragments were probed for reactivity with the 3B6 mAb by dot-blot assay. YIRTPACWD and full-length DTMUV E protein were used as negative and positive controls, respectively.

To determine the minimal epitope required for binding to the 3B6 mAb, we first expressed and purified several variants of the EXE/DPPFG fragment (Table 2). In a dot blot assay, we found that the 3B6 mAb recognized the EVE/DPPFG fragment variants and full-length E protein (Fig. 1) but not the control peptide YIRTPACWD. This result suggests that the EVE/DPPFG fragment is a B-cell epitope of the DTMUV E protein. To define the epitope more precisely, we next generated EXE/DPPFG fragments containing substitutions or C- or N-terminal deletions (Table 2). The V375A, V375L, or E376D substitutions did not abolish 3B6 mAb– binding activity (Fig. 1), suggesting that the ^375^X and ^376^E/D residues in the ^374^EXE/DPPFG^380^ epitope fragment are indiscriminate. In contrast, deletions of the ^374^E or ^380^G residues at the N or C terminus of ^374^EXE/DPPFG^380^ abolished 3B6 mAb binding, indicating that the ^374^EXE/DPPFG^380^ fragment is the minimal epitope mapped by the 3B6 mAb.

### Sequence analysis of the identified epitope in DTMUV and other flaviviruses

**Figure 2:**
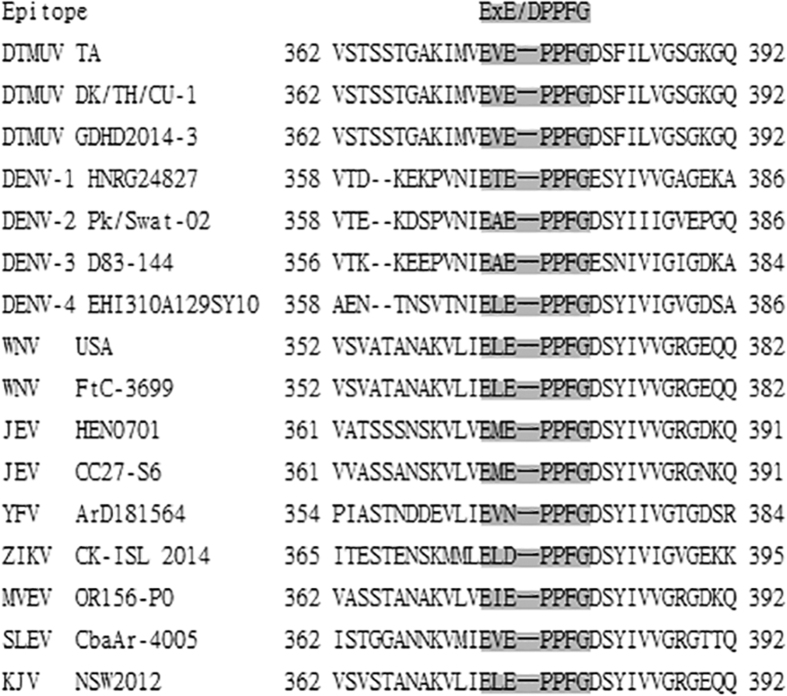
Sequence alignment of a minimal epitope in the E protein of flavivirus strains. The EXE/DPPFG epitope of various strains of DTMUV and other mosquito-borne flaviviruses were aligned using Lasergene software. Amino acid positions for each sequence are numbered on both sides. Dashes indicate identical amino acids. The identified epitope region is indicated by grey shading.

To determine whether the EXE/DPPFG sequence is conserved among the E proteins of DTMUV and other flaviviruses, we aligned the E protein amino acid sequences, including the EXE/DPPFG epitope region, of several flaviviruses. Specifically, we aligned the E protein sequences of three strains of DTMUV, four strains of DENV, two strains of WNV, two strains of JEV, ZIKV, yellow fever virus (YFV), Murray Valley encephalitis virus (MVEV), Saint Louis encephalitis virus (SLEV), and Kunjin virus (KJV) (Table 3). We found that the amino acids in the E protein minimal B-cell epitope of many mosquito-borne flaviviruses (Fig. 2) were essentially identical. The EXE/DPPFG motif was highly conserved (approximately 85%) in all the viruses tested, with variability occurring only in the indiscriminate ^375^X and ^376^E/D residues. The considerable sequence homology of the EXE/DPPFG motif suggests that it is a wide-spectrum epitope of many flaviviruses (Fig. 2).

### Epitope binding by duck DTMUV anti-serum

**Figure 3:**
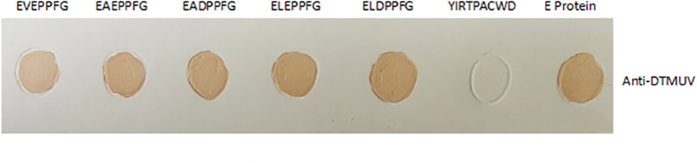
Identification of the E protein minimal epitope by DTMUV-positive duck serum. Peptide fragments were probed for reactivity with DTMUV-positive duck serum by dot-blot assay. YIRTPACWD and full-length E protein were used as negative and positive controls, respectively.

Dot-blot and western blot assays were used to test whether duck DTMUV anti-serum could recognize various EXE/DPPFG epitopes. Dot blots of EVEPPFG, EAEPPFG, EADPPFG, ELEPPFG, and ELDPPFG peptides, in addition to the full-length E protein, demonstrated positive reactivity to duck DTMUV anti-serum (Fig. 3), but the negative control peptide (i.e. YIRTPACWD) did not. This is further support that EXE/DPPFG most likely represents the minimal B-cell epitope of the DTMUV E protein, and the ^375^X and ^376^E/D residues are indiscriminate amino acids in these two positions. Western blot assays also confirmed the reactivity of the EXE/DPPFG epitope to duck DTMUV anti-serum (Supplementary Figure S2), suggesting that both methods reliably identify the minimal B-cell epitope of the DTMUV E protein.

### Epitope reactivity to ZIKV-, DENV-, JEV-, WNV-, and YFV-positive sera

**Figure 4:**
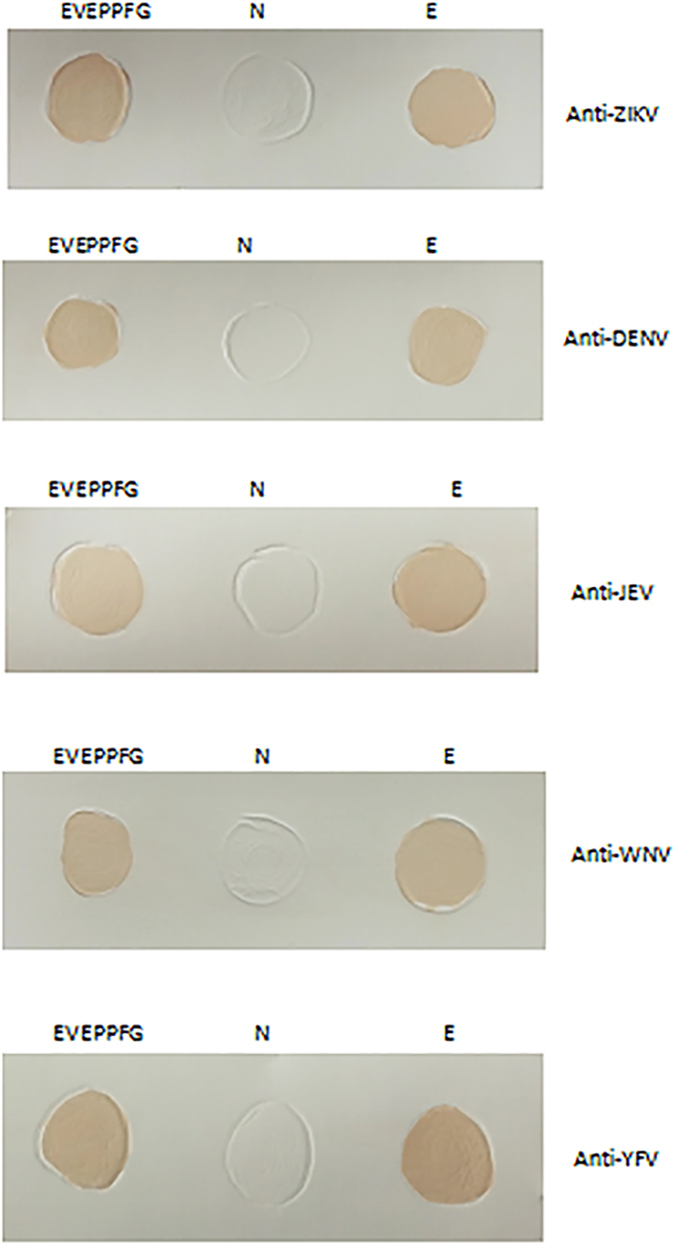
Cross-reactivity of the E protein minimal epitope with flavivirus-positive sera. The EVEPPFG epitope was probed for reactivity with various flavivirus-positive sera, including Zika virus (ZIKV), dengue virus (DENV), Japanese encephalitis virus (JEV), West Nile virus (WNV), and yellow fever virus (YFV), by dot-blot assay. YIRTPACWD (N) and full-length E protein (**E**) were used as negative and positive controls, respectively.

To determine the epitope cross-reactivity to other flavivirus, dot blots of the purified EVEPPFG fragment were incubated with ZIKV-, DENV-, JEV-, WNV-, and YFV-positive sera. All flavivirus-positive sera reacted with the EVEPPFG peptide and full-length E protein, but did not react with the negative control peptide (Fig. 4). Because the EXE/DPPFG epitope in the E proteins of MVEV, SLEV, and KJV is highly conserved (Table 3), it is likely that positive sera produced by these flaviviruses might also positively react. However, this could not be confirmed because MVEV-, SLEV-, and KJV-positive sera were unavailable.

### Location of the epitope on the E protein 3D structure

**Figure 5:**
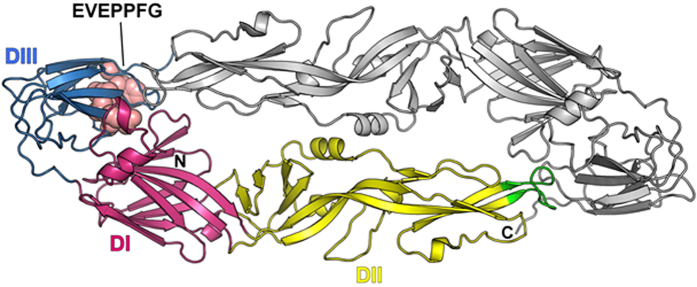
The EVEPPFG epitope of the DTMUV E protein is located in domain III. The protein-dimer structure of the DTMUV E protein was modeled using the crystal structure of the JEV E protein as a template. The domain I (DI), domain II (DII), and domain III (DIII) in one E protein monomer are coloured magenta, yellow, and blue, respectively. The other monomer is coloured grey. The location of the epitope is depicted as spheres and labeled. Two predicted N-glycosylation sites are coloured sky blue.

We evaluated the molecular structure and stereochemical quality of the DTMUV E protein with ProSA and PROCHECK. Using GlycoEP glycosylation prediction, we found that the DTMUV E protein most likely contains two N-glycosylation sites, ^154^N and ^314^N. Prediction scores for ^154^N and ^314^N were 0.838 and 0.438, respectively. However, using the NGlycPred algorithm to analyze the structural and residue pattern information of the epitope for potential N-glycosylation sites, we found that ^154^N, and not ^314^N, is likely the only site glycosylated. Modeling of the molecular structure of the DTMUV E protein revealed that the EXE/DPPFG epitope possesses a loop conformation and is located in the DIII domain but is not close to the DIII lateral ridge (Fig. 5).

## Discussion

Various mAbs have been used to identify flavivirus-specific epitopes and to investigate the antigenic structure of these epitopes. The DIII domain of the flavivirus E protein may play an important role in viral replication and infection. However, molecular information about the epitope in the DIII domain of the DTMUV E protein is lacking. The aim of this study was to investigate the antigenic site within the DIII domain using the E protein-specific 3B6 mAb. Using a 12-mer random peptides phage display system and mutagenesis, we precisely mapped a minimal B-cell epitope to amino acids 374 through 380 of the E protein. Duck DTMUV anti-serum positively reacted to the epitope, indicating the importance of these amino acids for antibody-epitope binding and the fact that detectable levels of antibody are generated to it following natural infection.

Sequence alignment revealed that the EXE/DPPFG epitope is highly conserved in DTMUV, ZIKV, WNV, DENV, MVEV, SLEV, KJV, and JEV, suggesting that the epitope has a similar function in these viruses. Cross-reactivity of ZIKV-, DENV-, JEV-, WNV-, and YFV-positive sera suggests that EXE/DPPFG is an immunodominant epitope among flaviviruses; however, the cross-reactivity of MVEV-, SLEV-, and KJV-positive sera is yet to be confirmed. Sequences alignments indicated that E of DTMUV showed 46.43-48.81% to DENV1-4, 61.48% to WNV, 64.87% to JEV, 43.65% to YFV, 64.47% to SLEV, 65.67% to KJV, 63.87% to MVEV, and 53.17% to ZIKV, but the EXE/DPPFG motif reacted to almost all flavivirus anti-serum, which suggests that the wide-spectrum epitope are unusual. In future vaccine development, it may be prudent to take effort to reduce induction of antibodies to this wide-spectrum immunodominant site to limit the formation of nonneutralized antibody complexes and antibody-dependent enhancement of the infection process. In contrast, design of optimal antigen peptides that specifically recognize the EXE/DPPFG epitope could improve the broad detection of DTMUV and other flaviviruses.

Using 3D-structure modeling of the DTMUV E protein, we observed that the EXE/DPPFG epitope is not located in the DII domain, as previously predicted^9^,^10^,^11^,^12^,^13^. In contrast, we found that the epitope is located in the DIII domain and does not protrude from the surface of the E protein. Previous studies have demonstrated that binding of some E reactive antibodies relies on the dynamic motion of protein molecules (i.e. “breathing”) in the virion particle, leading to transient exposure of buried epitopes^20^,^21^,^22^. Whether the 3B6 mAb requires such “breathing” for the epitope to become exposed to permit antibody binding remains to be resolved.

In conclusion, we have defined a novel epitope of the DTMUV E protein, its cross-reactivity to other flavivirus-positive sera, and its location within the 3D structure of the E protein. Our findings provide new insights into the structure and organization of the DTMUV E protein and valuable epitope information for the development of diagnostic assays and potential vaccines to prevent DTMUV and other flavivirus infections.

## Methods

### Generation of DTMUV virus, 3B6 mAb, and flavivirus-positive sera

DTMUV TA was grown in duck embryo fibroblasts cells or embryonated chicken eggs, as previously described^5^. Development and characterization of the E-specific 3B6 mAb has been described previously^23^. JEV- and WNV-positive rabbit sera were donated by Dr. Hua, and DENV-positive serum was donated by Dr. Qi Xian. ZIKV- and YFV-positive sera were obtained from the Human Zika Virus IgM ELISA Kit (MyBioSource) and the Human Yellow Fever Virus Antibody IgG ELISA Kit (MyBioSource), respectively.

### Affinity purification of the 3B6 mAb

The 3B6 mAb was purified from mouse ascites fluid using protein G agarose (Invitrogen), according to manufacturer instructions. The concentration of purified IgG was determined by measuring absorbance at 278 nm.

### Epitope mapping

The epitope was mapped with purified 3B6 mAb using the Ph.D-12^TM^ Phage Display Peptide Library Kit (New England BioLabs), as previously described^24^,^25^. Three rounds of biopanning were performed. Briefly, each well of a 96-well plate was coated with 10 μg/mL of purified 3B6 mAb and incubated with blocking buffer. The phage library was then added to the plate and incubated for 1 hour. After five washes with TBS buffer, 1 M Tris-HCl was added to the plate to elute the bound phages^24^,^25^. The phages were then amplified and titred on LB/IPTG/Xgal plates for selection. The ratio of output to input was calculated as the titre of the amplified output phages to the titre of the input phages.

### ELISA and sequencing of phage clones

After three rounds of biopanning, as described above and elsewhere^24^,^25^, individual phage clones were selected for target binding in an ELISA. Briefly, 96-well plates were coated with 100 ng of 3B6 mAb or mouse anti–porcine IFN-c (Sigma-Aldrich) as a negative control. The coated wells were then blocked, and the selected phages (10^10^ pfu/well, 100 μL/well) were added. The coated plates were then washed ten times with TBST, and bound phages were detected with horse radish peroxidase (HRP)-conjugated sheep anti–M13 antibody (Pharmacia), as described previously^24^,^25^. Colour development was achieved by adding a substrate solution containing *o*-phenylenediamine. Positive phage clones were sequenced as previously described^24^,^25^.

### Sequence analysis

To assess the level of conservation of the epitope among DTMUVs and other representative flaviviruses, we performed sequence alignments of the epitopes and their corresponding locations in the E protein of three DTMUV strains and several strains of other flaviviruses using Lasergene software (DNASTAR)^26^.

### Identification of the minimal epitope

To precisely define the minimal B-cell epitope of the DTMUV E protein, we designed and generated fragments corresponding to the roughly mapped epitope. Complementary oligonucleotide primers that were specific for each peptide fragment were designed as previously described^27^. Nucleotide segments with sticky ends were produced by *Eco*RI/*Xho*I digestion and direct annealing. The oligonucleotide fragments were then cloned into the pGEX6p-1 vector (GE Healthcare). The expressed peptides were purified using a GST Purification Kit (TaKaRa). Dot-blot assays were performed by spotting the purified peptide solutions onto a nitrocellulose membrane (Millipore). Approximately 1 μg of each synthesized peptide diluted with TNE buffer was spotted onto the membrane and incubated with the 3B6 mAb (diluted 1:2000 in PBS) or with duck DTMUV anti-serum (1:150 in PBS) at 37 °C for 1 hour. After washing three times with PBST, the membrane was probed with either HRP-conjugated goat anti–mouse IgG (1:500 dilution, KPL) or HRP-conjugated goat anti–duck IgG (1:500 dilution, KPL) at 37 °C for 1 hour. Western blots were performed by electrophoresis of purified GST-peptides in 10% polyacrylamide gels and transfer to nitrocellulose membranes. Membranes were incubated with duck anti–DTMUV serum diluted 1:150 in PBST, followed by reaction with HRP-conjugated goat anti–duck IgG (1:500 dilution, KPL) for 90 min at room temperature.

### Epitope cross-reactivity to ZIKV-, YFV-, WNV-, JEV-, and DENV-positive sera

Epitope cross-reactivity to sera infected with other flaviviruses was determined by dot blot, as described above. Briefly, approximately 1 μg of each synthesized epitope peptide diluted with TNE buffer was spotted onto a nitrocellulose membrane and incubated with ZIKV-, YFV-, WNV-, JEV-, and DENV-positive sera at 37 °C for 1 hour. After washing three times with PBST, the membrane was probed with a species-specific HRP-conjugated IgG (KPL) at 37 °C for 1 hour.

### Protein E modeling and prediction

To ascertain the location of the epitope within the 3D molecular structure of the DTMUV E protein, we performed *in silico* modeling. Because the DTMUV E protein shares 62% sequence identity with that of JEV, we used the crystal structure of JEV E protein (PDB ID: 3P54)^28^ as a modeling template using MODELLER software^29^. ProSA^30^ and PROCHECK^31^ software was used to validate the stereochemical quality of the final model. GlycoEP software^32^ and the NGlycPred algorithm^33^ were used to predict the N-glycosylation sites on the DTMUV E protein. The final structure was visualized and analyzed with PyMOL (v1.5.0.4, Schrodinger)^29^.

## Additional Information

**How to cite this article**: Li, C. *et al.* Identification of a New Broadly Cross-reactive Epitope within Domain III of the Duck Tembusu Virus E Protein. *Sci. Rep.* **6**, 36288; doi: 10.1038/srep36288 (2016).

**Publisher’s note:** Springer Nature remains neutral with regard to jurisdictional claims in published maps and institutional affiliations.

## Supplementary Material

Supplementary Information

## Figures and Tables

**Table 1:** Peptide sequences of selected phage clones.

Phage Clone	Sequence																			
1							V	S	**D**	Y	A	**F**	**G**	Y	W	N	T	L		
2							G	F	**D**	I	**P**	**F**	**G**	V	S	L	G	V		
3	G	S	F	A	M	K	**E**	L	Y	D	**P**	**F**								
4			Q	S	M	A	S	K	**D**	L	**P**	**F**	**G**	Q						
5					V	V	P	V	**D**	**P**	**P**	**F**	**G**	I	T	W				
6								D	**D**	V	**P**	**F**	**G**	R	N	P	A	M	G	
7	G	N	C	T	L	M	P	P	**D**	**P**	**P**	**F**								
8									E	E	**P**	**F**	**G**	T	F	V	D	R	M	A
9							**E**	A	S	Y	**P**	**F**	**G**	Y	F	A	T	A		
10							**E**	L	F	D	**P**	**F**	**G**	N	G	L	D	W		
11				A	I	S	W	T	**D**	V	**P**	**F**	**G**	S	K					
12				T	L	S	S	G	**E**	R	A	**F**	**G**	S	L					
13									**E**	**P**	A	**F**	**G**	L	P	P	M	A	Q	K
14						S	E	L	Y	K	**P**	**F**	**G**	T	F	R	H			
15	L	A	N	N	S	A	**E**	K	E	Y	**P**	**W**								
16	S	A	L	S	T	G	G	H	**D**	**P**	**P**	**F**								
17/18							H	P	N	V	**P**	**F**	**G**	V	I	A	D	G		
19							A	R	**D**	Y	**P**	**F**	**G**	V	Y	H	S	R		
20							**E**	A	S	Y	**P**	**F**	**G**	Y	F	A	T	A		
21									**D**	**P**	**P**	**F**	**G**	F	N	H	A	M	Y	
22	S	G	Q	A	T	S	**E**	V	L	**P**	**P**	**F**								
23	L	A	N	N	S	A	**E**	K	**E**	Y	**P**	P								
24				E	R	L	**E**	I	**E**	**P**	S	W	**G**	F						
25				N	L	G	G	K	**D**	**P**	L	**F**	**G**	I	Y					
26					L	A	N	R	**D**	**P**	A	**F**	**G**	S	L	S				
Consensus							**E**	X	**E/D**	**P**	**P**	**F**	**G**							
Virus TA		G	A	K	I	M	**E**	**V**	**E**	**P**	**P**	**F**	**G**	D	S	F				

Consensus amino acids are shown in bold.

**Table 2:** Primers for production of truncated peptides of the DTMUV E protein epitope.

Primer	Sequence	Truncated Peptide
3B6-1-F	5′-AATTCGAAGTGGAACCTCCATTCGGGC-3′	GST-EVEPPFG
3B6-1-R	5′-TCGAGCCCGAATGGAGGTTCCACTTCG-3′	
3B6-2-F	5′-AATTCGAAGTGGACCCTCCATTCGGGC-3′	GST-EVDPPFG
3B6-2-R	5′-TCGAGCCCGAATGGAGGGTCCACTTCG-3′	
3B6-3-F	5′-AATTCGAAGTGGAACCTCCATTCC-3′	GST-EVEPPF
3B6-3-R	5′-TCGAGGAATGGAGGTTCCACTTCG-3′	
3B6-4-F	5′-AATTCGTGGAACCTCCATTCGGGC-3′	GST-VEPPFG
3B6-4-R	5′-TCGAGCCCGAATGGAGGTTCCACG-3′	
3B6-5-F	5′-AATTCGAAGCAGAACCTCCATTCGGGC-3′	GST-EAEPPFG
3B6-5-R	5′-TCGAGCCCGAATGGAGGTTCTGCTTCG-3′	
3B6-6-F	5′-AATTCGAAGCAGACCCTCCATTCGGGC-3′	GST-EADPPFG
3B6-6-R	5′-TCGAGCCCGAATGGAGGGTCTGCTTCG-3′	
3B6-7-F	5′-AATTCGAACTGGAACCTCCATTCGGGC-3′	GST-ELEPPFG
3B6-7-R	5′-TCGAGCCCGAATGGAGGTTCCAGTTCG-3′	
3B6-8-F	5′-AATTCGAACTGGACCCTCCATTCGGGC-3′	GST-ELDPPFG
3B6-8-R	5′-TCGAGCCCGAATGGAGGGTCCAGTTCG-3′	

**Table 3:** Flavivirus strains used in sequence analysis.

Species	Strain	GenBank No	Site	Year of Isolation
DTMUV	TA	JQ289550.1	China	2010
DTMUV	DK/TH/CU-1	KR061333.1	Thailand	2013
DTMUV	GDHD2014-3	KT159713.1	China	2014
DENV-1	HNRG24827	KC692511.1	Argentina	2010
DENV-2	DENV-2/Pk/Swat-02	KJ701507.1	Pakistan	2013
DENV-3	D83-144	KJ737430.1	Thailand	1983
DENV-4	EHI310A129SY10	JX024758.1	Singapore	2010
WNV	USA	AY646354.1	USA	2002
WNV	FtC-3699	KR868734.1	USA	2012
JEV	HEN0701	FJ495189.1	China	2007
JEV	CC27-S6	AY303797.1	Taiwan	2003
YFV	ArD181564	JX898880.1	Senegal	2005
ZIKV	CK-ISL 2014	KJ634273.1	Cook Islands	2014
MVEV	OR156-P0	KC852193.1	Australia	2012
SLEV	CbaAr-4005	FJ753286.2	Argentina	2005
KJV	NSW2012	KT934804.1	Australia	2012
