# Supplementary material for: Identification of a New Broadly Cross-reactive Epitope within Domain III of the Duck Tembusu Virus E Protein
Source: Sci Rep. 2016 Nov 8;6:36288. doi: 10.1038/srep36288 (PMC5099753; doi:10.1038/srep36288)
Supplement: Supplementary Information [file srep36288-s1.pdf]

Identification of a New Broadly Cross-reactive Epitope within Domain III of the Duck Tembusu Virus E Protein

Chenxi Li[1], Xiaofei Bai[1], Runze Meng[1], Wulin Shaozhou [1], Qingshan Zhang[1], Ronghong Hua[1], Jyung-Hurng Liu[2], Ming Liu[1, *], Yun Zhang[1,*]

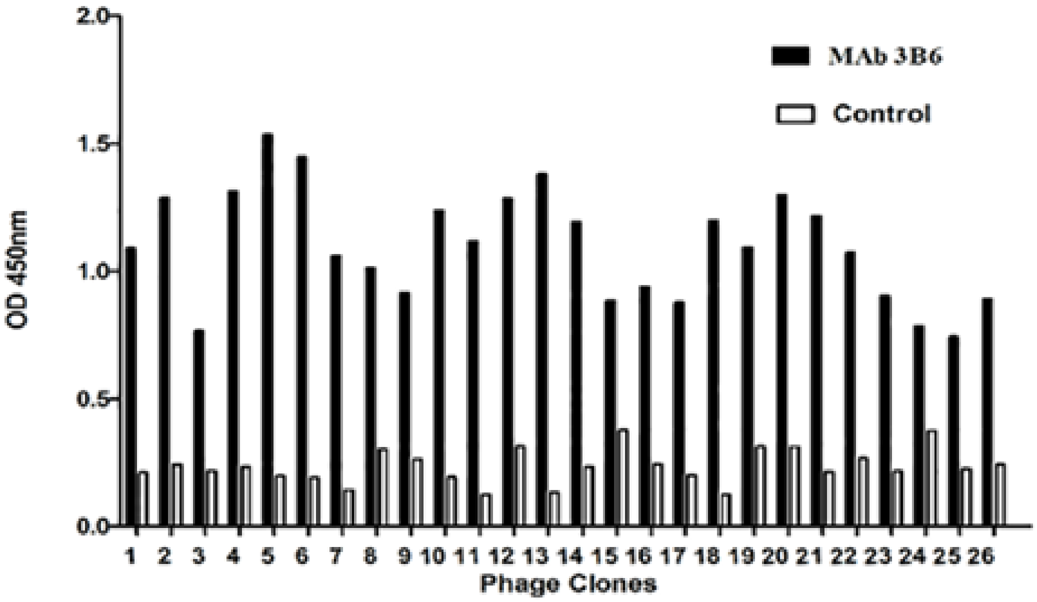

Figure S1. Detection of selected phages for mAb 3B6 bining by Phage ELISA. Selected phage clones after three rounds of biopanning were detected by mAb 3B6 or by the anti-porcine IFN-c mAb (negative control).

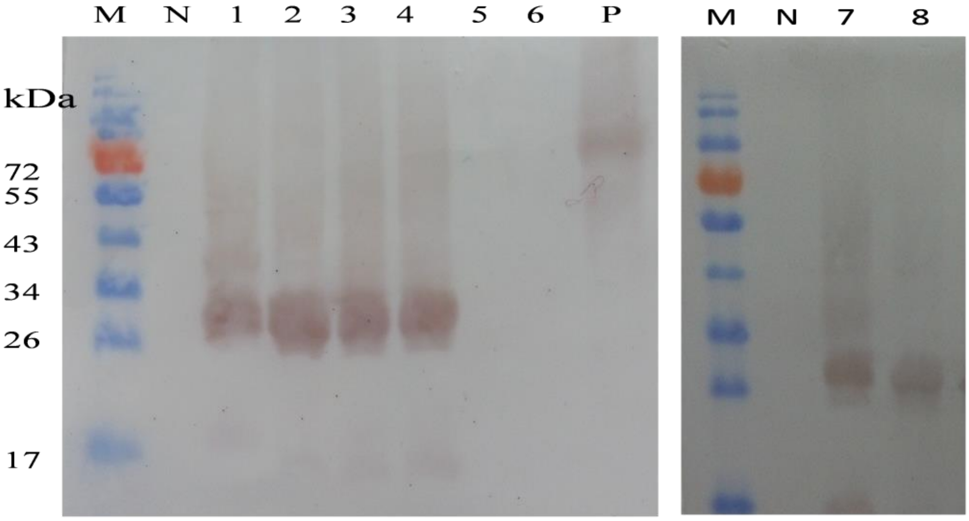

Figure S2. The reactivity of the synthesized peptides to duck anti-DTMUV serum by Western blot analysis. YIRTPACWD and E protein were used as negative (N) and positive (P) control, respectively. M, Protein marker; 1, EVEPPFG; 2, EVDPPFG; 3, EAEPPFG; 4, EADPPFG; 5, VEPPFG; 6, EVEPPF; 7, ELEPPFG; 8, ELDPPFG.
